# Osteomalacia associated with cutaneous psoriasis as the presenting feature of coeliac disease: a case report

**Published:** 2012-03-27

**Authors:** Faten Frikha, Mouna Snoussi, Zouhir Bahloul

**Affiliations:** 1 Department of Internal Medicine, Hospital of Hedi Chaker, 3029 Sfax, Tunisia

**Keywords:** Osteomalacia, cutaneous psoriasis, celiac disease, Vitamin D, Bone loss, gluten-free diet, Tunisia

## Abstract

Celiac disease (CD) is a chronic digestive disease that results in hypersensitivity to the gliadin fraction of Gluten. Malabsorption syndrome may be responsible for weight loss, diarrhea, osteomalacia, and vitamins deficiency. Herein we report a patient with coeliac disease (CD) who presented with osteomalacia and psoriasis without classical symptoms of CD. A 25-year-old North African Tunisian white woman was admitted to the hospital because of a 1-year history of bone pain, weight loss and weakness. She had cutaneous psoriasis on dermatologic examination. She had also anemia, hypocalcemia and pathological fracture. She was diagnosed to have osteomalacia on the basis of clinical, biological and radiological findings. Further investigations revealed the presence of antiglutaminase antibodies, and histopathologic findings of the duodenal biopsy were consistent with celiac disease. The patient showed a fast response to gluten-free diet, and full recovery with calcium and vitamin D replacement. Coeliac disease is frequently misdiagnosed leading to major complications such as osteolamacia. In the other hand, osteomalacia can still be the presenting feature of undiagnosed celiac disease. Association between osteomalacia and cutaneous psoriasis is rarely reported.

## Background

Celiac disease (CD) is an autoimmune inflammatory disease of the small intestine precipitated by the ingestion of gluten, in genetically susceptible persons [1]. Classically, it manifests with intestinal symptoms (diarrhea, steatorrhoea, abdominal pain or discomfort) associated with weight loss and anemia. Besides the intestinal symptomatology, the disease is often accompanied by extra intestinal complications including osteopenia or osteoporosis and osteomalacia [2]. There are very few reports of osteomalacia as the presenting symptom of CD [1,3]. Herein we report a patient with undiagnosed coeliac disease (CD) who presented with osteomalacia and cutaneous psoriasis without classical symptoms of CD.

## Patient and case report

A 25 year-old North African Tunisian white woman with unremarkable medical history, presented to our department of Internal Medicine with erythematic and squamous cutaneous lesions associated to bone pain, weakness and important weight loss. She complained of a diffuse bone pain which has progressed gradually over the last 2 years to the point that she had difficulties in rising from a chair and walking.

On physical examination, she had pale conjunctive and showed a waddling gait disturbance. Her right hip range of motion was limited and painful. Her weight was 36 Kg and her height was 160 cm. She denied any gastrointestinal symptoms. The dermatologic examination noted a dry erythrodermis to hick squamous with nail psoriatic changes called Beau lines (Lines going across the nails (side to side rather than root to tip). Laboratory tests revealed microcytic anemia (haemoglobin at 9.6 g/dl and mean corpuscular volume at 77 fl), severe hypocalcemia (corrected serum calcium level at 1.84 mmol/l), mild hypophosphoremia (0.6 mmol/l) and raised alkaline phosphatase (606 UI/l). Other laboratory data were normal including erythrocyte sedimentation rate (ESR), serum creatine kinase and liver enzymes. Renal function was within normal value. Work-up disclosed low 25-hydroxy vitamin D at 4 ng/l (normal 30-100 ng/l) and high parathormone at 264 pg/ml (normal 8.7-79 pg/ml). The 24 hour urinary calcium was low at 0.28 mmol/day.

**Figure 1:**
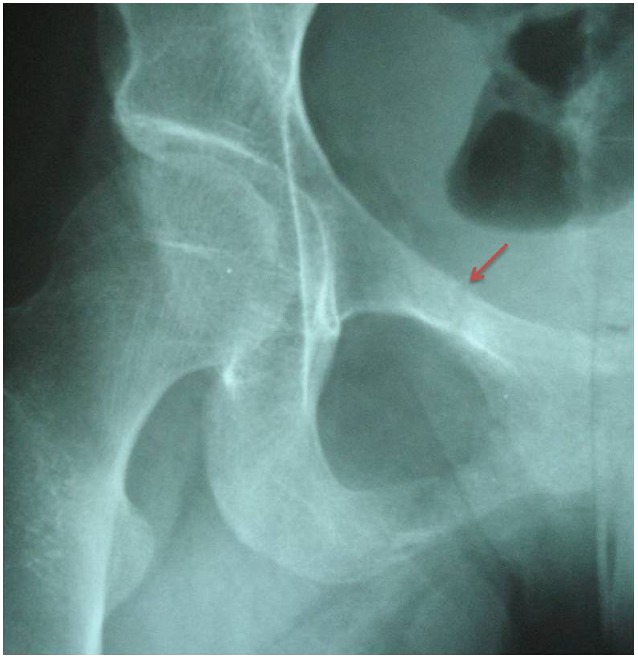
Radiograph of right hip showing fracture with Loozer's zones of the ilio-pubic rami

**Figure 2:**
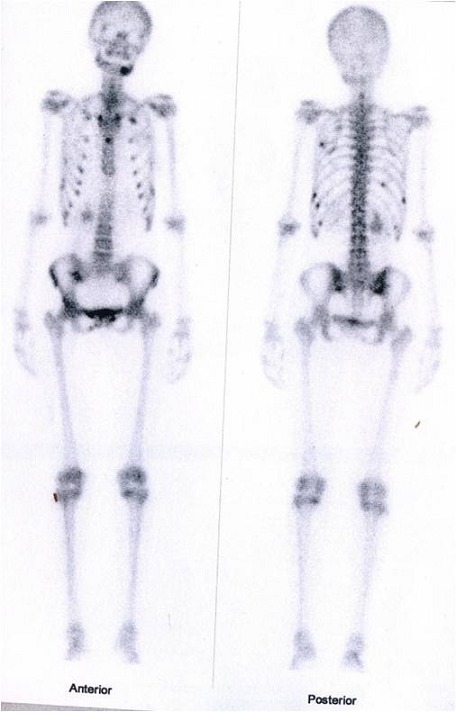
Whole body bone scan: multiple hot spots on rib cage, sternum and pelvis (ilium, and sacrum)

X-ray examination of the hip revealed a fracture of the right ilio-pubic rami (Figure 1). The Technetium 99m- whole-body bone scintigraphy evidenced multiple hot spots on the rib cage, sternum and pelvis (Figure 2). These images were consistent with osteomalacia. Dual-energy X-ray absorptiometry (DEXA) scan was performed. Bone mineral density revealed markedly low density at both lumbar vertebra (z-score at -3.5 SD) and femoral neck (z-score at -3.6 SD). The diagnosis of osteomalacia was made by clinical, biochemical and radiological features, and investigations for malabsorption were carried out. Antiglutaminase antibodies were strongly positive (titre>140 UI/l). Esophagogastroduodenal endoscopic examination with distal duodenal biopsy revealed diffuse villous atrophy accompanied by mononuclear infiltrates consistent with the diagnosis of celiac disease. Treatment with calcium supplementation (1 g per day) and vitamin D was prescribed associated with a gluten free diet. At check-up one month later, the patient had improved markedly with a complete resolution of her symptoms.

### Consent

Written informed consent was obtained from our patient for publication of this case report and accompanying images. A copy of the written consent is available for our patient.

## Discussion

Celiac disease (CD) is a chronic digestive disease that results in hypersensitivity to the gliadin fraction of Gluten. Classically, the disease manifests with diarrhea, weight loss and anemia. Its Diagnosis is based on positivity of IgA and IgG antigliadin and endomysial antibodies and characteristic endoscopic detection of inflammation and atrophy of duodenal mucosa [2]. Women comprise approximately 75 % of newly diagnosed adult coeliac disease cases and they tend to have more clinically prominent disease [1]. The disease is often accompanied by extraintestinal complications including anemia, dermatitis herpetiformis, depression, dementia, dental enamel defects, osteopenia or osteoporosis and osteomalacia [1,4].

This patient presented with severe osteomalacia in the absence of any gastrointestinal symptoms such as diarrhoea, steatorrhoea or abdominal discomfort. An association between celiac disease and osteomalacia was first reported in 1953 [5]. It was reported in few cases that osteomalacia could be the presenting feature of coeliac disease [3–14]. Although, two large series of celiac patients have reported no evidence of osteomalacia [15,16], many other authors emphasize that CD must be suspected in patients with osteomalacia [8,17]. The disease is considered as the second most common cause of osteomalacia after gastrectomy in the United States [1]. Osteomalacia in adults, like in our patient, starts insidiously with pains in the lumbar region and thighs, spreading later to the arms and ribs. Patients show often an extreme weakness, and there is difficulty in climbing up stairs and getting up from a squatting position.

Hypocalcaemia in CD- as in this case- is caused by reduced intestinal calcium absorption as a result of vitamin D deficiency. It is also due to reduced absorptive area secondary to villous atrophy [18]. The mechanism of development of bone pathology in those patients with untreated celiac disease is not fully defined, but chronic negative calcium balance and vitamin D deficiency are the potential factors. Bone loss is explained by the overproduction of cytokines IL-1 alpha, IL-1 beta and TNF-alpha which is further accelerated by hyperparathyroidism connected with malabsorption of calcium and vitamin D. Interaction of both these mechanisms increases bone resorption and activates bone loss.

Some studies of metabolic bone disease in celiac disease showed that osteomalacia, tetany, and bone fracture can be the presenting symptoms of coeliac disease, especially when the malabsorption is florid [11]. However, the occult form of CD seen in our case, which is commonly seen in the elderly, may be associated with a risk of osteoporosis or osteomalacia related fractures, similarly as the classic symptomatic disease [2].

We emphasize that the diagnosis of coeliac disease should be suspected in any patient with osteomalacia [17]. On the other hand, detailed analysis of calcium metabolism, including markers of bone remodelling and X-ray densitometry (DXA), are recommended in all patients with verified celiac disease [1,2]. Screening with bone densitometry is helpful in detecting patients at risk of fracture [19,20] and the presence of severely reduced bone mineral density is associated with fracture at these sites.

A combination of gluten-free diet and vitamin D supplementation is almost an effective mode of therapy [17] and lead in almost cases to rapid resolution of all symptoms. Our case presented with classical signs of osteomalacia, complicated by secondary hyperparathyroidism, and associated with cutaneous changing. She showed an excellent response to gluten-free diet and oral vitamin D treatment.

Finally, and even the association between osteomalacia and cutaneous psoriasis was rarely reported [18], we would like to underline that outside of its action on the phosphocalcic metabolism, the vitamin D acts on the skin, especially on the proliferation of the keratinocytes that allowed its indication in the treatment of the psoriasis [18].

## Conclusion

Osteomalacia remains a common complication of coeliac disease and may occur even without clinical or a biochemical abnormality of CD as it was shown by our case. Treatment with a gluten-free diet associated with Calcium and vitamin D supplementation lead to resolution of symptoms and can dramatically improve bone density.
